# *Linum aksehirense* (sect. Dasylinum, Linaceae), a new species from Central Anatolia (Turkey)

**DOI:** 10.3897/phytokeys.136.46477

**Published:** 2019-12-10

**Authors:** Osman Tugay, Deniz Ulukuş

**Affiliations:** 1 Selçuk University, Department of Pharmaceutical Botany, Faculty of Pharmacy, Selçuklu, Konya, Turkey Selçuk University Konya Turkey; 2 Selçuk University, Department of Biotechnology, Faculty of Sciences, Selçuklu, Konya, Turkey Selçuk University Konya Turkey

**Keywords:** Endemic, Konya, Linaceae, *
Linum
*, taxonomy

## Abstract

*Linum aksehirense* (Linaceae) is described as a new species known from the slopes of the Sultan Mountains in the Akşehir district of Konya in Central Anatolia (Turkey). It is most similar to *L. pubescens* Banks & Sol. and *L. anisocalyx* P.H.Davis, from which it is easily distinguished by its stem leaf shape, sepal shape and petal colour. Seed and pollen surface ornamentations were photographed under SEM microscopy to explore micromorphological characters distinguishing the new species from close relatives. In addition, photographs of living material, a distribution map, ecological details, and an identification key are provided.

## Introduction

The genus *Linum* Linnaeus (Linaceae) is comprised of about 200 species in the Linaceae family. *Linum* is distributed mainly in North America, the Balkan Peninsula, Anatolia, as well as in Eurasia and Africa (Robertson 1971), but it is also represented in South America and Australasia.

*Linum* was first described by Linnaeus (1753). The first comprehensive study on the genus was made by Planchon (1847, 1848). The most recent worldwide treatment of *Linum* was done by Winkler (1931). According to Winkler (1931) and Planchon (1847, 1848), *Linum* is divided into five sections. The genus has been the subject of several taxonomic studies and regional revisions, such as those focusing on American and South African *Linum* species (Rogers 1963, 1981; Mildner and Rogers 1978), as well as Flora Europaea (Ockendon and Walters 1968), Flora of the U.S.S.R. (Yuzepchuk 1974), and Flora of Turkey and the Aegean Islands (Davis 1967). The genus plays an important role in the economic and social development of humans. For example, the seeds of *L. usitatissimum* are used nutritionally and medicinally. Linseed oil is also a significant source of inks, varnishes, and lubricants (McDill et al. 2009).

*Linum* is represented by four sections in Turkey: sect. SyllinumGrisebach (1843: 115), sect. LinastrumPlanchon (1847: 597), sect. DasylinumPlanchon (1847: 598), and sect. LinumPlanchon (1847: 598). With 54 taxa (Yılmaz 2018), Turkey is one of the most important centres of genetic diversity for the genus *Linum*. The new species described in this paper brings the number to 55 taxa in Turkey, 26 (47%) of which are endemic.

Linum sect. Dasylinum is characterised by having perennial or annual life cycles and distinct morphological characteristics. Leaves are alternate, often hairy. Petals usually have coherent claws and are blue, pink or white. Capsules are hairy or glabrous (Davis 1967). There have been numerous studies on the morphology of *Linum* in Turkey by several authors (Davis 1967; Yılmaz and Kaynak 2008, 2010; Yılmaz 2010). There have also been investigations on a worldwide scale concerning the palynology (Erdtman 1969; Rogers and Xavier 1971; Saad 1961, 1962; Xavier et al. 1980; Rogers 1985; Talebi et al. 2012), karyology (Ray 1944; Harris 1968; Rogers et al. 1972) and anatomy (Winkler 1931; Metcalfe and Chalk 1950) of the genus. There has been just one study on *Linum* seeds, which covers several species of the genus (Özcan and Zorlu 2009).

From a biogeographical and evolutionary point of view, McDill et al. (2009) reported that section Linum and *Dasylinum* were not monophyletic and that *Linum* appears to have arisen in Eurasia, from which it spread to Africa, North America, South America and Australasia.

We collected samples of what we suspected could be a new species of *Linum* while conducting field work around the Sultan Mountains between 2011 and 2017. The specimens were checked against the Flora of Turkey and the East Aegean Islands, as well as neighbouring floras (Flora Iranica, Flora Iraq, Flora of the U.S.S.R. and Flora Europaea). We subsequently decided that it was a species new to science.

The aim of this study was to describe the new species, named *Linum aksehirense*, occurring in the Sultan Mountains (Akşehir, Konya), in terms of its morphology, palynology and seed micromorphology.

## Material and methods

Field collections were carried out in the Sultan Mountains from 2011 to 2017. Collected specimens were dried using traditional procedures and were deposited in the KNYA Herbarium. They were identified using the Flora of Turkey and the East Aegean Islands using stereo microscopy and checking them against other *Linum* accounts found in relevant literature. The taxonomical description of the species was made according to Davis (1967).

For palynological investigations, pollen slides were prepared according to Wodehouse’s (1935) technique. The pollen micromorphology of *L. aksehirense* was scrutinized by scanning electron microscopy (SEM) techniques. For SEM, pollen grains were directly placed on double-sided carbon tape affixed to aluminium stubs, covered with gold with a Hummle VII sputter coater and photographed at magnifications of 2000× to 7000× with a JEOL-5600. SEM micrographs were used to determine exine sculpturing of the pollen. Pollen terminology was based on Punt et al. (2007). For seed investigations, SEM micrographs were used to determine seed coat sculpturing. The terminology of Stearn (1983) and Özcan and Zorlu (2009) was adopted to describe the SEM aspects of the seed coat.

## Taxonomy

### Linum aksehirense

Taxon classificationPlantaeMalpighialesLinaceae

Tugay & Ulukuş sp. nov.

2D5FD121-031E-548B-84DE-29CF33704CC5

urn:lsid:ipni.org:names:77203431-1

**Figure 1.**
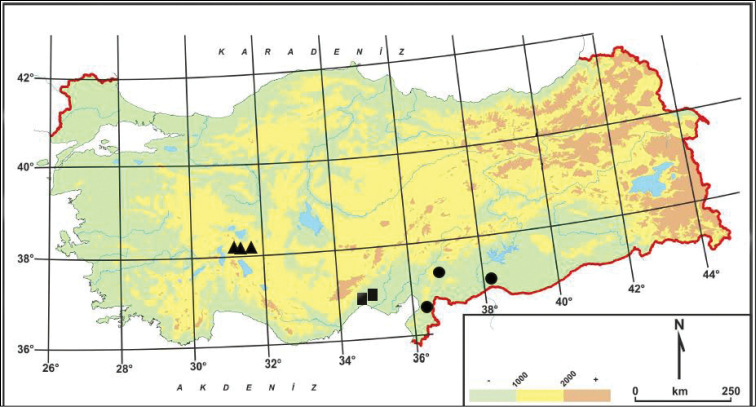
Distribution map of *Linum aksehirense* (triangle), and closely related *Linum pubescens* (circle) and *Linum anisocalyx* (square) in Turkey.

**Figure 2.**
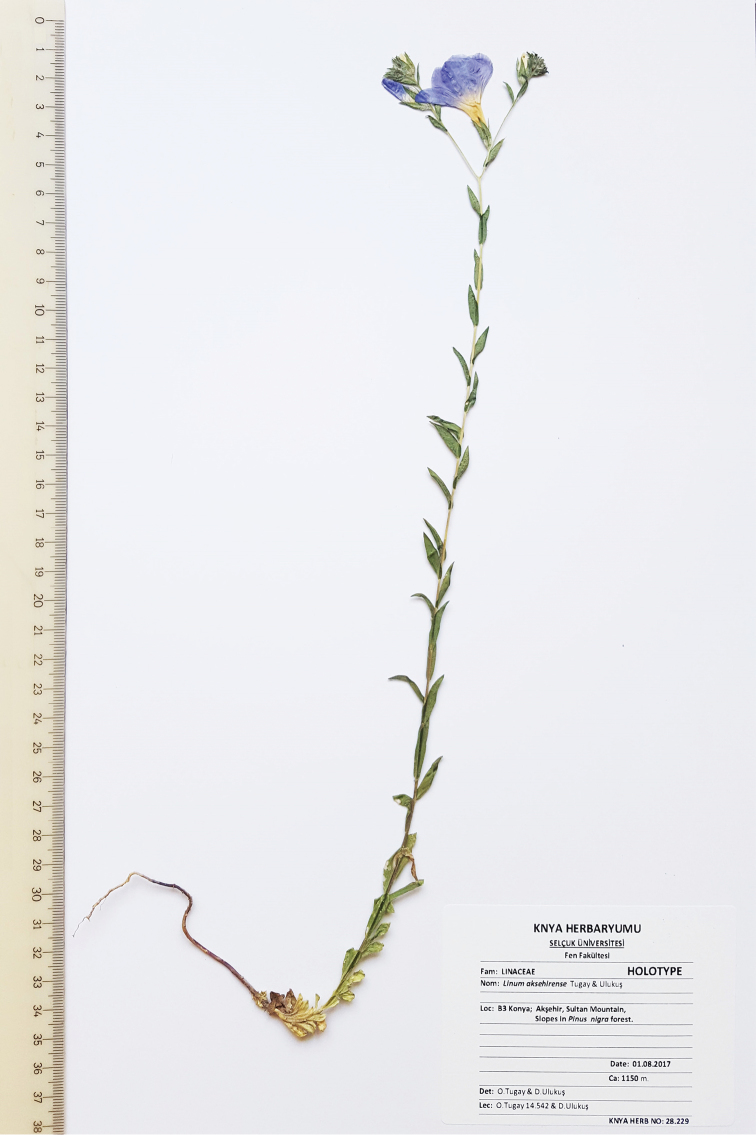
Holotype specimen of *Linum aksehirense* Tugay & Ulukuş.

**Figure 3.**
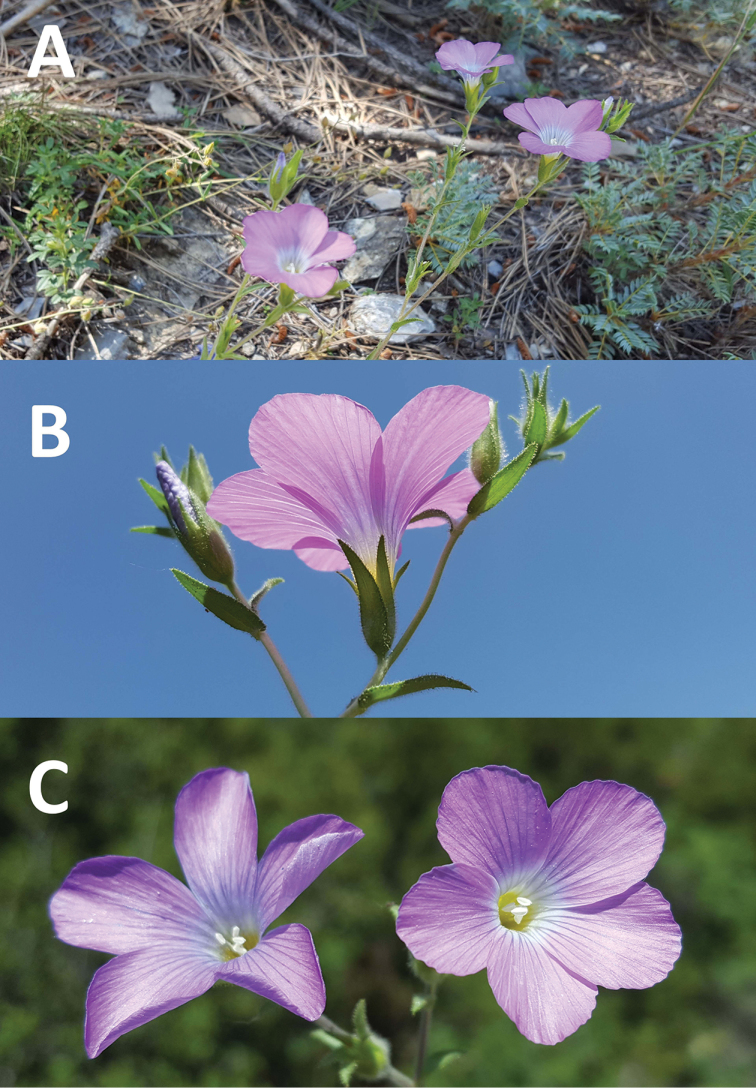
General view of habit and flower **A–C***Linum aksehirense*.

**Figure 4.**
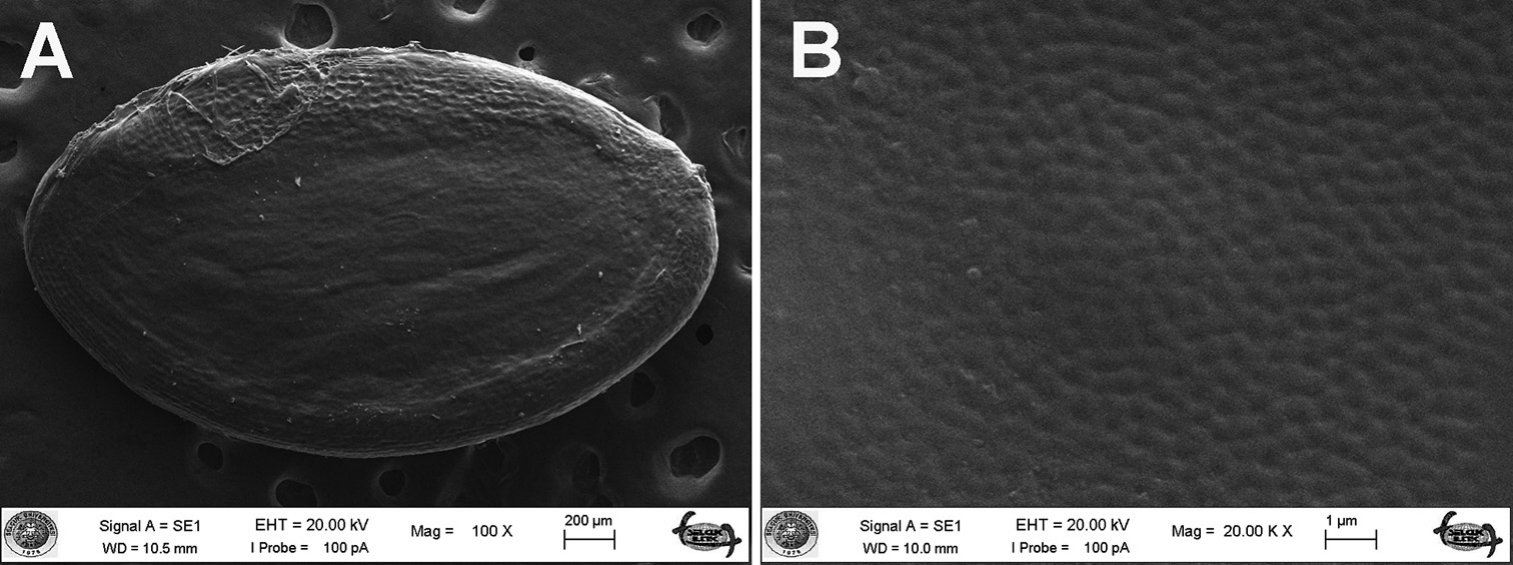
Scanning electron micrographs of seeds *Linum aksehirense* species **A** general view and **B** surface sculpturing pattern.

**Figure 5.**
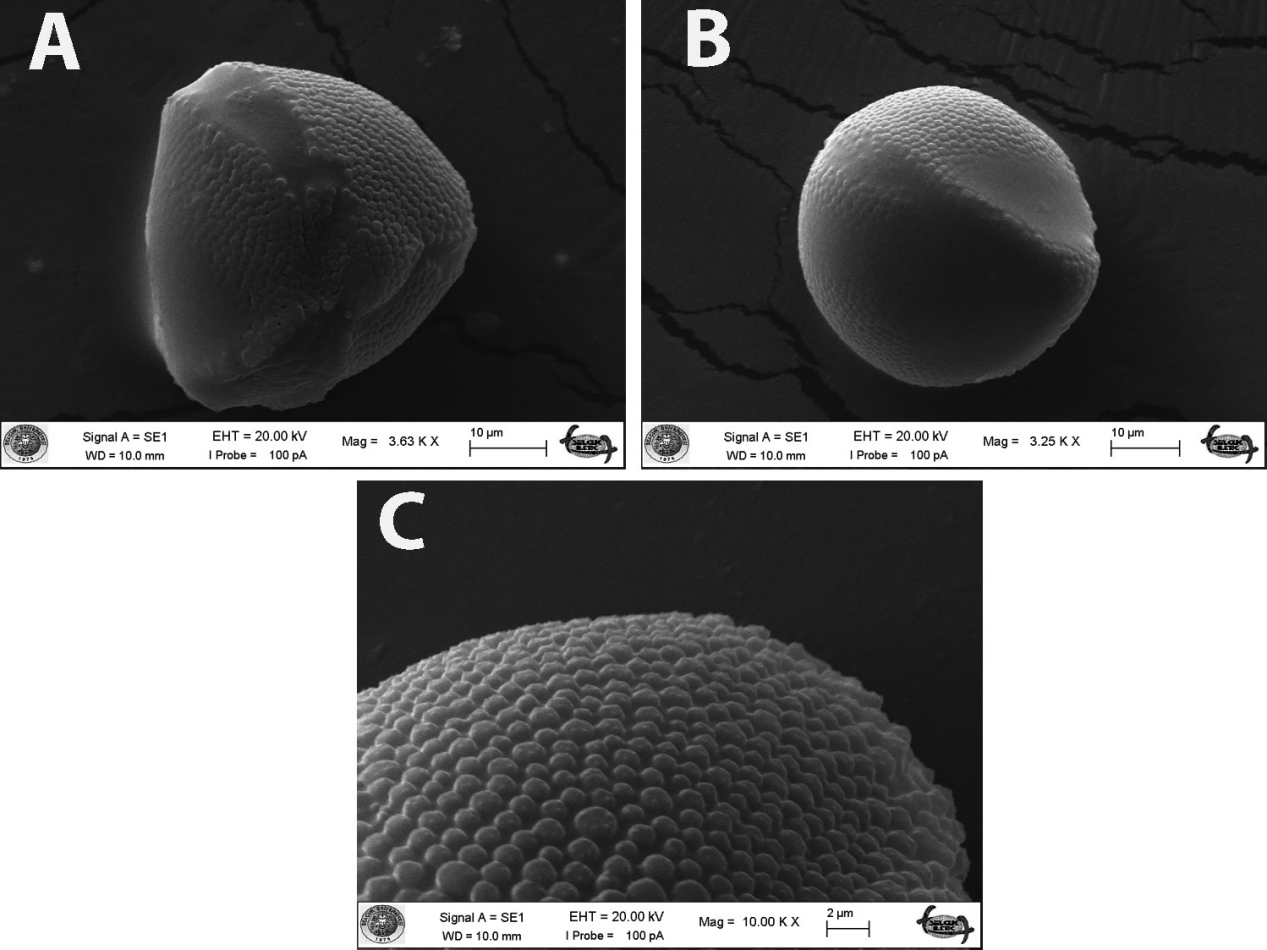
SEM micrographs of the pollen grains of *L. aksehirense***A–C** polar, equatorial view and ornamentation (*O.Tugay* 14.542 & *D.Ulukuş*).

**Figure 6.**
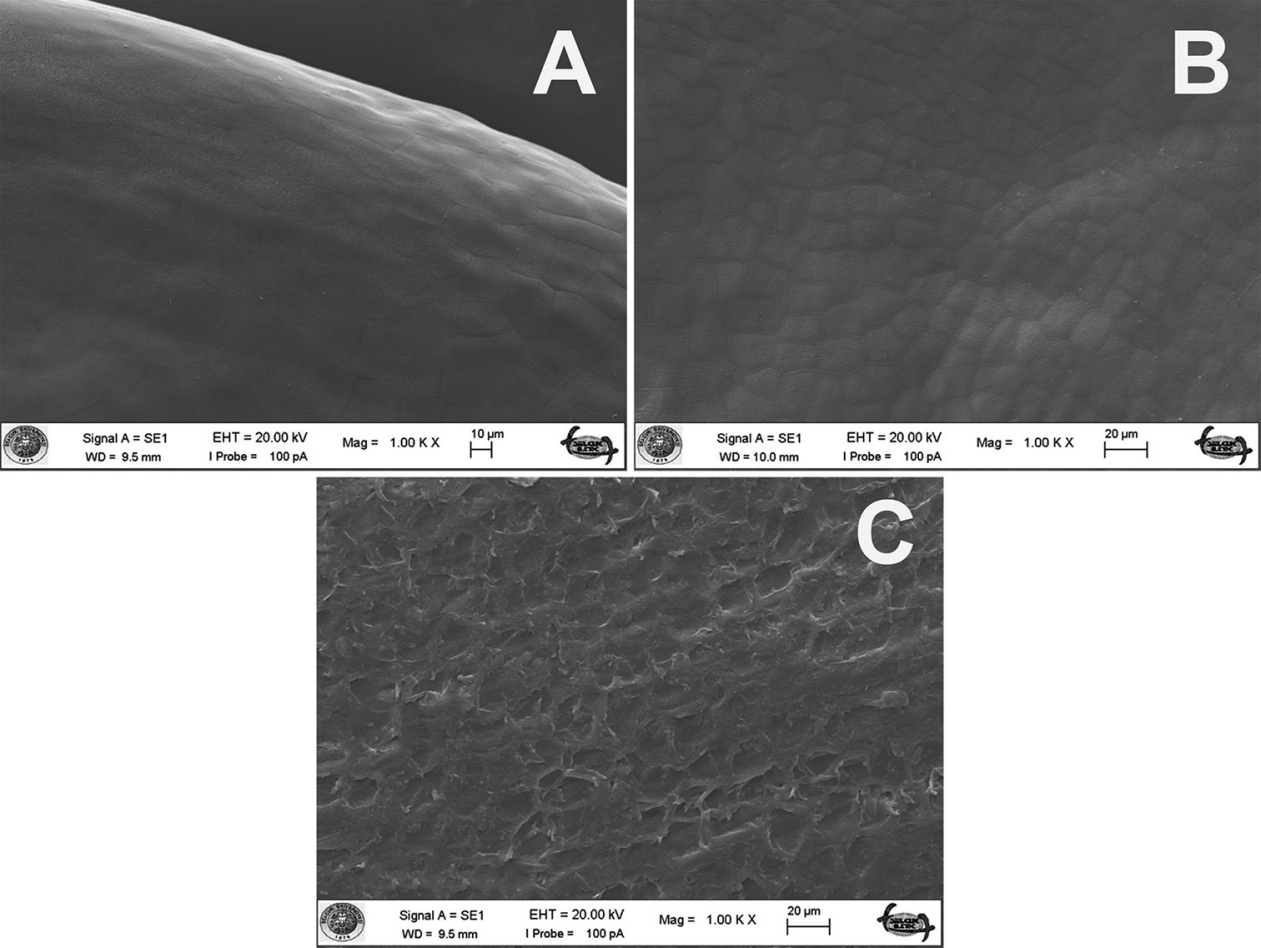
Scanning electron micrographs of seeds sculpturing patterns *Linum* species **A***L. aksehirense***B***L. anisocalyx***C***L. pubescens*.

Figures 1 , 2 , 3 , 4 , 5 , 6

#### Diagnosis.

*Linum aksehirense* belongs to Linum sect. Dasylinum. The new species is similar to *L. pubescens* but is distinguished by its spathulate lower stem leaves that are not evanescent (*vs.* oblong-spathulate evanescent), subequal lanceolate sepal shape (*vs.* subequal linear), petals 27–33 mm (*vs.* 18–27 mm) and petals that are blue-violet with a yellowish limb base (*vs.* pink with a bluish limb base).

#### Type.

Turkey. B3 Konya; Akşehir, Sultan Mountains, slopes in *Pinus nigra* forest, 1150 m alt., 38°19.230'N, 31°23.181'E, 01 August 2017, *O.Tugay* 14.542 & *D.Ulukuş* (holotype KNYA, isotypes KNYA 28.229).

#### Description.

The plant is a green annual. Flowering stems villous, erect, 22–33 cm; sterile shoots few. Lower stem leaves spathulate, not evanescent, 10–13 × 2–4 mm; median stem leaves lanceolate-elliptic, acute, 16–19 × 2–3.5 mm, 1–3 nerved. Bract (similar to median stem leaves) stipitate glandular margined, lanceolate, 14–15 × 3–3.5 mm. Cymes divaricate, lax, few-flowered; flowers 3–7 per stem; pedicels 1–2 mm, not elongated in fruit. Sepals subequal, lanceolate, 12–14 × 2–3 mm, bearing long hairs and stiputate glandular margined. Petals blue-violet (blue when dry) with a yellowish base to the limb, 27–33 mm. Capsule 5 × 5 mm diam. with 1–1.5 mm beak. Seeds elliptic-oblong, 2–2.5 × 1–1.5 mm, brown, glossy. Sculpturing of seed coat is reticulate-rugolose-ruminate (Fig. 4A–B).

**Paratypes.** Turkey. B3 Konya; Akşehir, slopes, 1170 m alt., 38°20'N, 31°24'E, 31 August 2011, *O.Tugay* 7.182 (KNYA); Akşehir, Hıdırlık, slopes, 1100 m alt., 38°19'N, 31°23'E, 01 August 2017, *O.Tugay* 14.520 & *D.Ulukuş* (KNYA).

#### Ecology.

*Linum aksehirense*, which is endemic to Turkey, grows between 1100 and 1170 m elevation on slopes with *Pinus nigra* forest. The vegetation of this habitat is composed mainly of herbaceous and suffruticose plants including: Agrimonia eupatoria L. subsp. asiatica, Campanula lyrata Lam. subsp. lyrata, *Centaurea virgata* Lam., *Cistus laurifolius* L., Cota tinctoria (L.) J.Gay ex Guss. var. tinctoria, Digitalis ferruginea L. subsp. ferruginea, Dianthus crinitus Sm. var. crinitus, Hedysarum varium Willd. subsp. varium, *Phlomis armeniaca* Willd., Pinus nigra J.F.Arnold subsp. nigra, Prunus divaricata Ledeb. var. divaricata, *Scabiosa rotata* M.Bieb., *Securigera varia* (L.) Lassen, Teucrium chamaedrys L. subsp. chamaedrys and *Teucrium polium* L.

#### Phenology.

The new species was observed flowering in July and collected fruiting from July to August.

#### Etymology.

The species epithet comes from ‘Akşehir’, where the new species is found.

#### Proposed Turkish name for the new species.

Akşehir keteni.

#### Distribution and conservation status.

*Linum aksehirense* is known from three localities in Konya province, in the Irano-Turanian phytogeographic region (Fig. 1). Its area of occupancy is estimated to be less than 500 km^2^. The number of mature individual plants is estimated to be less than 250. Being an annual gives this new species a crucial advantage for survival against potential threats, which include the destruction of brush by locals, road construction and deterioration of habitat. Thus, according to criterion B and D, it can be included in the EN (Endangered) category (IUCN 2001; 2016).

##### Key to the species of Linum sect. Dasylinum in Turkey

**Table:** 

1	Annuals	**2**
–	Perennials	**5**
2	Petals c. 8 mm, free; sepals not or slightly longer than capsule	***L. seljukorum***
–	Petals 18 mm or more; coherent; sepals much longer than capsule	**3**
3	Petals 27–33 mm, blue-violet with a yellowish base to the limb	***L. aksehirense***
–	Petals 18–27 mm, pink with a bluish base to the limb	**4**
4	Sepals subequal	***L. pubescens***
–	Sepals very unequal, the outer two hiding the much shorter inner sepals	***L. anisocalyx***
5	Median cauline leaves margined by stalked glands	**6**
–	Median cauline leaves not margined by glands	**8**
6	Inflorescence compact; petal claw 1/4 as long as limb	***L. densiflorum***
–	Inflorescence widely spreading, or rarely 1–3 flowered	**7**
7	Petal claw c. ½ as long as limb; median stem leaves not attenuate below	***L. hirsutum***
–	Petal claw c. 1–1½ as long as limb; median stem leaves attenuate at both ends	***L. unquiculatum***
8	Plants very twiggy at base, with many sterile shoots; 1–7 flowers	***L. olympicum***
–	Plant herbaceous, with few or no sterile shoots; usually > 7 flowers	**9**
9	Cymes usually spreading, lax; leaves 1–3 nerved, oblong, linear or subspathulate; petal claw c. ½ as long as limb	***L. hirsutum***
–	Cymes compact; leaves 3–7 nerved, broadly lanceolate; petal claw ¼–1/3 as long as limb	***L. hypericifolium***

##### Pollen morphology

The pollen shape of the new species was subprolate (P/E: 1.13) with a polar axis of 53.85 ± 1.75 µm (mean ± standard deviation) μm and an equatorial axis of 47.70 ± 4.70 μm. The aperture was tricolpate. The colpus was long-acute ended with a colpus length of 34.82 ± 3.26 μm and width of 12.91 ± 2.23 μm. Exine thickness was 0.5 μm and intine thickness was 0.4 μm. Exine ornamentation was densely gemmate (Fig. 5 A–C).

## Discussion

*Linum aksehirense* is similar to *L. pubescens*, *L. anisocalyx* and *L. viscosum* in morphology. However, it differs from these similar species in several vegetative and reproductive characters (Table 1).

**Table 1.** Morphological comparison of *Linum aksehirense*, *L. pubescens*, *L. anisocalyx* and *L. viscosum*.

**Characters**	***L. aksehirense***	***L. pubescens***	***L. anisocalyx***	***L. viscosum***
Stem	unbranched or branched at the upper stem	branched at the median stem	branched at the base	unbranched or branched at the upper stem
Lower stem leaf shape	spathulate not evanescent	oblong-spathulate evanescent	oblong-spathulate evanescent	lanceolate, ovate-lanceolate evanescent
Median stem leaf shape	lanceolate-elliptic, acute, 16–19 × 3–4 mm	oblong, subacute 12–23 × 2–5 mm	oblong, subacute 12–23 × 2–5 mm	lanceolate, subacute 14–21 × 3–5 mm
Sepal shape	subequal lanceolate	subequal linear	very unequal elliptic-lanceolate	subequal lanceolate
Sepal size (mm)	12–14 × 2–3	9–12 × 1–2	outer sepals 11–12 × 3–3.5; inner sepals 6–8 × 2.5–3	6–9 × 1–1.5
Petal colour	blue-violet with a yellowish base to the limb	pink with a bluish base to the limb	pink with a bluish base to the limb	pink
Petal size (mm)	27–33	18–27	18–26	16–21

According to Davis (1967), *L. anisocalyx* is closely related to *L. pubescens*, differing from *L. pubescens* primarily by its strongly dimorphic sepals (not dimorphic), which are rhomboid-lanceolate and glandular-margined.

*Linum aksehirense* differs from *L. pubescens* by its spatulate, not evanescent lower stem leaves (*vs.* oblong-spathulate evanescent), lanceolate-elliptic, acute median stem leaves, 16–19 × 3–4 mm (*vs.* oblong, subacute 12–23 × 2–5 mm), subequal lanceolate sepals (*vs.* subequal linear), sepal size of 12–14 × 2–3 mm (*vs.* 9–12 × 1–2 mm) and its petal colour, which is blue-violet with a yellowish limb base (*vs.* pink with a bluish limb base) (Table 1).

*Linum aksehirense* is similar to *L. anisocalyx*, differing in its lanceolate-elliptic, acute median stem leaves, 16–19 × 3–4 mm (*vs.* oblong, oblong, subacute 12–23 × 2–5 mm), subequal lanceolate sepals (*vs.* very unequal elliptic-lanceolate), sepal size of 12–14 × 2–3 mm (*vs.* outer sepals 11–12 × 3–3.5 mm, inner sepals 6–8 × 2.5–3 mm), petal colour, which blue-violet with a yellowish limb base (*vs.* pink with a bluish limb base) and in its petal size, which is 27–33 mm (*vs.* 18–26 mm) (Table 1).

*Linum aksehirense* can be distinguished from *L. viscosum*, by its spathulate not evanescent (*vs.* lanceolate, ovate-lanceolate, evanescent), sepal size of 12–14 × 2–3 mm (*vs.* 6–9 × 1–1.5 mm), petal colour, which is blue-violet with a yellowish limb base (*vs.* pink), and in its petal size, which is 27–33 mm (*vs.* 18–21 mm).

Xavier et al. (1980) described the basic pollen grain in *Linum* as subspheroidal, about 50 μm in diameter, isopolar, radially symmetric, tricolpate, colpi with pointed ends. However, in our study *L. aksehirense* had subprolate pollen shape. According to Talebi et al. (2012)’s palynologic study on four section of the Linaceae, including 15 taxa of *Linum*, in all examined taxa the pollen shape in polar view was circular (except in *L. densifolorum* where it was concave-triangular) and also the exine sculpturing pattern showed a clavate, pilate and gemmate to baculate form. Talebi et al. (2012) reported that pollen features of *L. densifolorum* consisted of an oblate-spheroidal pollen shape and small and large gemmate exine ornamentation. Our findings showed that *L. aksehirense* had subprolate pollen shape and the exine ornamentation was densely gemmate (Fig. 5 A–C). In terms of pollen micromorphology, palynological results demonstrated that there are no clear differences among species of the same section. However, pollen shape can be used to distinguish species.

Özcan and Zorlu (2009) studied seed surface patterns *Linum* genus and found reticulate-ruminate patterns in sect. Dasylinum. Özcan and Zorlu (2009) showed that seed patterns provide characters to distinguish taxa at specific and infraspecific levels. In this study, the micromorphological study of the seeds showed that there were clear differences among the studied species. According to our findings, *Linum aksehirense* had reticulate-rugulose-ruminate sculpturing while *L. pubescens* exhibited reticulate-rugulose-granulate and *L. anisocalyx* showed reticulate-rugulose (Fig. 6 A–C).

## Conclusion

With the discovery of this new species, the number of species of *Linum* in Turkey has risen to 55. This study provides material and data to aid further research on this significant genus of the Linaceae.

## Supplementary Material

XML Treatment for Linum aksehirense

## Appendix I

Additional examined specimens

***Linum pubescens***; C5/6 Turkey, Hatay: near sea level, rocky limestone slopes, 07 May 1965, *Coode* 620 & *Jones* (E!); C5/6 Syria/Turkey, Hatay: nr. Aleppo, or between there and Iskenderun, *Russell* (BM photo!); C6 Gaziantep: 30 km. S of Aintab, *Dinsmore* 8.935!; Urfa: Rum Kala’a, *Sint.* 1888: 268!; C8 Mardin: 11 km. W of Idil, 800 m, 05 May 1966, *Davis* 42.427 (E!).

***Linum anisocalyx***; C5 Içel: plaine de Mersina, May 1855, *Balansa* (holo. K!); Içel: Mersin, 1896, *Siehe* 199 (E!); Içel: Mersin to Kuzucubelen, 18 June 1950, 500 m, *Hub.-Mor.* 10.567!

***Linum viscosum***; Spain, Gerona: Roadside near Ripoll, 13 July 1979, *Rogers* 13.567 (B photo!), Huesca, near Jaca, 8 July 1979, *Rogers* 13.558 (B photo!); Austria: Ebersberg en Autriche. In campis aridis Sabulosis, 1812 (GDC photo!); Italy: Gênes, 08 July 1808, Candolle, (GDC photo!).
